# Latent Profiles and Network Characteristics of Alexithymia Among Undergraduate Nursing Students

**DOI:** 10.1155/da/8930217

**Published:** 2026-11-15

**Authors:** Ying Li, Songwei Zhong, Meng Xiang

**Affiliations:** ^1^ College of Sports Science Jishou University Jishou Hunan China jsu.edu.cn; ^2^ School of Physical Education and Arts Hunan University of Medicine Huaihua Hunan China hnucm.edu.cn

**Keywords:** alexithymia, childhood trauma, mental health, nursing education

## Abstract

**Background:**

Alexithymia is prevalent among nursing students and significantly impairs their psychological well‐being. While the overall burden of this condition is well‐documented, prior studies have predominantly focused on variable‐centered prevalence rates, overlooking the latent heterogeneity of alexithymic traits in this population. This study employed latent profile analysis (LPA) to examine the heterogeneity of alexithymia among undergraduate nursing students by identifying subgroups with distinct symptom patterns. Subsequently, factors associated with subgroup membership were explored, and symptom network characteristics were analyzed within each latent profile.

**Methods:**

This cross‐sectional study recruited 1434 undergraduate nursing students from 10 Chinese universities using an online convenience sampling strategy. Participants completed an electronic survey assessing sociodemographic characteristics, alexithymia, childhood trauma (CT), and physical activity. Data were analyzed using LPA to identify subgroups, multinomial logistic regression to determine predictors, and network analysis to map symptom interconnections.

**Results:**

LPA identified three distinct subgroups: low alexithymia group (42.259%), moderate alexithymia group (26.569%), and hyperalexithymia group (31.172%). According to the multinomial logistic regression analysis, female gender, CT, and low professional interest were identified as the primary predictors of higher alexithymia risk. Crucially, network analysis revealed different central nodes across the profiles. Specifically, difficulty identifying feelings (Toronto Alexithymia Scale [TAS]‐II) was the central hub in the moderate alexithymia group, while lack of fantasy (TAS‐III) was the core node in the hyperalexithymia group.

**Conclusion:**

Alexithymia in undergraduate nursing students exhibits substantial heterogeneity, manifesting as three distinct profiles. By identifying the predictors and central symptoms of each subgroup, this study provides an empirical foundation for targeted psychological support. These findings can help nursing educators implement tailored interventions to address the specific vulnerabilities of each profile.

## 1. Introduction

Alexithymia is fundamentally characterized by deficits in emotional expression, restricted imaginative capacity, and limited emotional experience [1]. Individuals with this trait struggle to comprehend their own and others’ emotions and face significant challenges in regulating and processing affective states [2, 3]. Epidemiologically, the prevalence of alexithymia ranges from 10% to 13% in the general population [4], surges to 36.6% among Chinese adolescents [5], and remains notably high within university student cohorts [6]. Extensive research indicates that alexithymia predisposes individuals to a spectrum of adverse outcomes, including emotional distress (e.g., depression, loneliness, and boredom proneness) [7], interpersonal difficulties (e.g., social anxiety and avoidance), and maladaptive behaviors such as fear of missing out (FoMO), social aggression, and eating disorders [8]. Consequently, these individuals frequently exhibit constrained emotional expression, impaired social functioning, and diminished psychological adaptability [9, 10].

As the cornerstone of the healthcare system, nurses play an irreplaceable role in patient care, clinical observation, treatment execution, and health promotion. Their psychological well‐being directly dictates professional efficacy, service quality, and ultimately, patient safety and clinical outcomes [11]. The presence of alexithymia in nursing professionals significantly compromises their occupational performance and mental health, dampening work motivation, impairing crisis response capabilities, and deteriorating nurse–patient communication. Over time, the accumulation of such psychological strain can degrade the overall quality of care, posing tangible risks to patient safety [12]. As the future backbone of the nursing workforce, it is imperative for nursing students to maintain robust psychological well‐being to sustain healthcare system development and elevate service standards [13]. However, alexithymia has been recognized as an underlying psychological vulnerability among nursing students and has been associated with an increased risk of both somatic and psychiatric disorders [14]. Therefore, greater attention should be paid to alexithymia and psychological well‐being among nursing students. According to Yao et al. [15], when conceptualized within Chinese culture, alexithymia manifests as a multidimensional framework comprising three core features: impaired emotional identification and description, a scarcity of fantasy, and an externally focused cognitive style.

Historically, research assessing alexithymia among nursing students has predominantly relied on aggregate total scale scores [16]. However, this variable‐centered approach is inherently limited; students with identical total scores may exhibit fundamentally distinct symptomatic patterns across individual items. Such methodological oversimplification obscures individual heterogeneity, frequently resulting in inefficient and imprecise management strategies [17, 18]. To overcome this limitation, stratifying individuals into homogeneous subgroups enables the delivery of tailored interventions, thereby maximizing therapeutic efficacy. Latent profile analysis (LPA), a robust person‐centered statistical method, addresses this gap by classifying individuals into distinct latent subgroups based on continuous observed variables [19]. By optimizing the identification of unique profile characteristics and highlighting significant intergroup differences, LPA provides a rigorous empirical foundation for designing highly personalized intervention protocols [19].

Furthermore, precisely delineating the core dimensions of alexithymia is critical as these serve as the primary focal points for clinical intervention. Given the multidimensional nature of the construct, prioritizing these central components allows for the optimal allocation of resources and enhanced intervention efficiency. Consequently, a granular examination of the internal structure of alexithymia is indispensable. Network analysis emerges as an innovative methodological framework that utilizes both visual and quantitative techniques to intricately map the complex interplay among various alexithymia dimensions [20]. According to network theory, nodes exhibiting high centrality indices exert substantial influence within the system, rendering them optimal targets for therapeutic intervention. Strategically targeting these central nodes is likely to generate cascading effects, thereby alleviating symptoms across peripheral dimensions [21]. Moreover, integrating external factors that influence alexithymia in undergraduate nursing students into the network model not only elucidates underlying structural pathways but also establishes a robust theoretical foundation for developing highly precise and effective intervention strategies, ultimately providing actionable insights for future clinical practice and intervention design [22]. This study had three primary objectives: first, to identify distinct subgroups of undergraduate nursing students with alexithymia using LPA; second, to examine the factors associated with different alexithymia subgroups; and third, to explore the core features and symptom networks of alexithymia within each subgroup by applying network analysis. The findings are expected to provide valuable insights for developing targeted interventions for alexithymia in this population.

## 2. Materials and Methods

### 2.1. Participants

A convenience sample of undergraduate nursing students was obtained from 10 higher education institutions across Shandong, Hubei, Hunan, and Henan provinces between April and June 2025. Participants completed an online questionnaire administered through an electronic survey platform. On the first page of the survey, the study objectives were clearly outlined, and an informed consent procedure was implemented. Participants were required to click “agree” to proceed with the questionnaire, whereas those who declined were automatically exited from the survey. To ensure anonymity, the links to the electronic questionnaire were distributed to the students by their respective class instructors. Data collection continued until no new responses were received for seven consecutive days. A total of 1446 questionnaires were initially collected. Following data cleaning, 12 invalid questionnaires were excluded due to substantial missing data or obvious regular response patterns. Ultimately, 1434 valid questionnaires were included in the statistical analysis.

1. Inclusion criteria were as follows: (1) full‐time undergraduate nursing students (2); fluent in Mandarin Chinese (3); absence of reading impairments; and (4) provision of informed consent and voluntary participation in the study.
2. Exclusion criteria included (1) presence of major psychiatric disorders, in accordance with the Declaration of Helsinki to protect vulnerable populations, (2) currently receiving psychotherapy or taking antipsychotic medications, and (3) being on a medical or academic leave of absence during the study period.

### 2.2. Measures

#### 2.2.1. Social Demographic Information

Sociodemographic characteristics, encompassing gender, age, family residence, and monthly household income, were collected from the participants.

#### 2.2.2. The Childhood Trauma (CT) Questionnaire—Short Form (CTQ‐SF)

The CTQ‐SF, originally developed by Bernstein et al. [23], is a 28‐item self‐report measure utilized to evaluate five distinct domains of childhood maltreatment (with three items excluded from the subscale scoring). These items are evenly distributed across five subscales: physical abuse, emotional abuse, sexual abuse, emotional neglect, and physical neglect, with each domain containing five items. Responses are recorded on a five‐point Likert scale ranging from 1 (“never”) to 5 (“always”). Prior to statistical analysis, reverse scoring must be applied to items 2, 5, 7, 13, 19, 26, and 28. Individual subscale scores span from 5 to 25, yielding a total possible score between 25 and 125, where elevated scores reflect a greater severity of CT exposure. The cutoff scores for identifying moderate to severe levels of CT were defined as follows: emotional neglect ≥ 15, emotional abuse ≥ 13, physical neglect ≥ 10, physical abuse ≥ 10, and sexual abuse ≥ 8 [24]. In the present study, the total scale exhibited robust internal consistency, yielding a Cronbach’s alpha coefficient of 0.893.

### 2.3. Toronto Alexithymia Scale (TAS‐26)

TAS‐26 was originally developed by Bagby et al. [25] and subsequently translated into Chinese by Yao et al. [15]. This 26‐item instrument evaluates four core dimensions: difficulty describing feelings, difficulty identifying feelings, externally oriented thinking, and fantasy. Items are rated on a five‐point Likert scale ranging from 1 (“strongly disagree”) to 5 (“strongly agree”). Elevated total scores reflect a greater severity of alexithymia. In the current study, the scale exhibited satisfactory internal consistency, with a Cronbach’s α coefficient of 0.882 for the total scale.

### 2.4. Physical Activity Rating Scale

Participants’ physical activity levels over the past week were assessed using PARS‐3, originally established by Liang [26]. The scale assesses physical activity by evaluating three dimensions: exercise intensity, exercise frequency, and exercise duration. These aspects are evaluated using a five‐point rating system, with intensity and frequency scored from 1 to 5, and duration ranging from 0 to 4. The overall physical activity score is computed as the product of these three dimension scores, yielding a total possible score between 0 and 100. Higher total scores denote greater engagement in physical activity.

### 2.5. Statistical Analysis

Data processing and statistical modeling were executed utilizing Mplus (Version 8.3), IBM SPSS Statistics (Version 26.0), and the R computing environment. Baseline characteristics were summarized via descriptive statistics, with continuous data reported as means accompanied by standard deviations and categorical data expressed as absolute frequencies alongside percentages. To uncover unobserved heterogeneity in alexithymia profiles among the nursing cohort, LPA was applied to the item‐level responses of the assessment scale [27]. The comparative fit of competing models was appraised via the Akaike information criterion (AIC), Bayesian information criterion (BIC), and sample‐size‐adjusted BIC (aBIC), wherein diminished metrics denote superior model adequacy. Profile separation and classification precision were quantified by the entropy index, with estimates approaching unity, reflecting optimal categorization accuracy. The optimal number of latent classes was determined by comparing each k‐class model with the corresponding *k* − 1 class model using the bootstrapped likelihood ratio test (BLRT) and the Lo–Mendell–Rubin adjusted likelihood ratio test (LMRT). A significant *p*‐value in these tests substantiates the retention of the more complex *k*‐class structure [28, 29]. Intergroup differences regarding categorical covariates were examined via chi‐square tests. Covariates demonstrating statistical significance in these preliminary screenings were subsequently incorporated into a multinomial logistic regression framework to extract independent predictive factors. For the network construction, the qgraph and bootnet packages in R were employed to map inter‐variable associations by estimating a Gaussian Graphical Model (GGM). To optimize model selection and prevent overfitting, edge weights were regularized via the graphical least absolute shrinkage and selection operator (gLASSO), guided by the extended BIC (EBIC) with a tuning parameter of *γ* = 0.5. In terms of node centrality, the strength index was prioritized; this metric quantifies the cumulative absolute weight of all edges linked to a specific node, thereby reflecting its overarching influence within the network topology. Finally, the robustness of the edge weights and centrality metrics was assessed via a non‐parametric bootstrapping procedure comprising 1500 iterations. The correlation stability (CS) coefficient was also computed to quantify index reliability, wherein a CS value exceeding 0.25 was deemed acceptable and a value above 0.50 indicated robust stability [30].

## 3. Results

### 3.1. Participant Characteristics

The study included 1434 undergraduate nursing students aged 17–26 years. The sample was predominantly female (80.4%). Comprehensive sociodemographic characteristics of the participants are detailed in Table 1.

**Table 1:** Demographic and academic‐related characteristics by latent profiles (*N* = 1434).

Variable	*N* (100%)	Low alexithymia (606)	Moderate alexithymia (381)	Hyperalexithymia (447)	*χ* ^2^/*Z*	*p*
Gender	—	—	—	—	17.594	<0.001
Male	281 (19.6)	148	53	80	—	—
Female	1153 (80.4)	458	328	367	—	—
Age	—	—	—	—	22.652	<0.001
≤20 year	652 (45.5)	290	135	227	—	—
21–23 year	678 (47.3)	277	210	191	—	—
>23 year	104 (7.2)	39	36	29	—	—
Only child	—	—	—	—	10.582	0.005
Yes	313 (21.8)	147	92	74	—	—
No	1121 (78.2)	459	289	373	—	—
Family residence	—	—	—	—	5.961	0.051
Urban	687 (47.9)	288	201	198	—	—
Rural	747 (52.1)	318	180	249	—	—
Hold a position	—	—	—	—	3.353	0.763
Student union	103 (7.2)	41	27	35	—	—
Club officer	80 (5.6)	33	20	27	—	—
Class committee member	318 (22.2)	126	83	109	—	—
No	933 (65.1)	406	251	276	—	—
Monthly household income (¥)	—	—	—	—	21.951	0.001
≤3000	178 (12.4)	94	35	49	—	—
3001–6000	558 (38.9)	227	132	199	—	—
6001–9000	399 (27.8)	168	116	115	—	—
≥9001	299 (20.9)	117	98	84	—	—
Clinical practice	—	—	—	—	31.815	<0.001
No	1039 (72.5)	466	234	339	—	—
Yes	395 (27.5)	140	147	108	—	—
Family atmosphere	—	—	—	—	29.978	<0.001
Highly democratic	512 (35.7)	198	175	139	—	—
Moderately democratic	823 (57.4)	357	194	272	—	—
Authoritarian	99 (6.9)	51	12	36	—	—
Nursing interest	—	—	—	—	41.805	<0.001
Dislike	242 (16.9)	95	99	48	—	—
Neutral	1065 (74.3)	451	264	350	—	—
Like very much	127 (8.9)	60	18	49	—	—
Personality	—	—	—	—	24.307	<0.001
Introverted	233 (16.2)	81	85	67	—	—
Neutral	859 (59.9)	371	232	256	—	—
Extroverted	342 (23.8)	154	64	124	—	—
Childhood trauma	—	37.470 ± 12.242	29.837 ± 5.930	35.910 ± 11.217	63.537	<0.001
Physical activity	—	17.172 ± 16.366	19.249 ± 16.798	16.096 ± 16.227	3.875	0.021

### 3.2. LPA of Alexithymia

LPA was performed using the 26 items of the TAS‐26 as indicator variables to explore the latent heterogeneity of alexithymia among undergraduate nursing students. Models specifying one to five latent profiles were subsequently estimated and compared (Table 2 and Figure 1). As the profile solution increased from one to five, the AIC, BIC, and aBIC exhibited a continuous downward trend. However, the five‐profile solution was discarded because the LMRT failed to reach statistical significance (*p* > 0.05). The three‐profile solution yielded an entropy value of 0.917. Although slightly lower than that of other models, this value exceeded 0.90, indicating robust classification accuracy. Moreover, the three‐class model offered enhanced theoretical interpretability and a clearer delineation of alexithymia features across the student population. Consequently, the three‐profile model was selected as the best‐fitting solution. The three extracted subgroups were designated as the Low alexithymia group (42.259%), Moderate alexithymia group (26.569%), and Hyperalexithymia group (31.172%).

**Figure 1:**
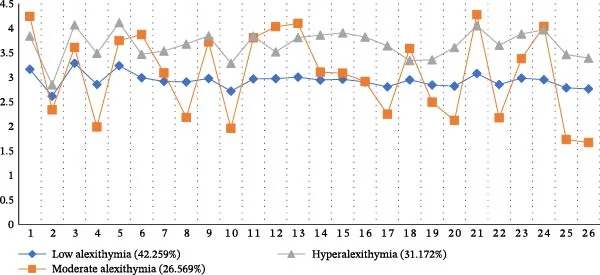
Estimated sample item means for the three latent profiles of alexithymia.

**Table 2:** Model fit indices for latent profile analysis (*N* = 1434).

Indices	Unconditional model				
	One‐profile	Two‐profile	Three‐profile	Four‐profile	Five‐profile
Fit statistics					
AIC	99,751.753	94,991.873	**91,561.277**	88,753.112	87,698.270
BIC	100,025.701	95,408.063	**92,119.709**	89,453.786	88,541.186
aBIC	99,860.514	95,157.106	**91,782.983**	89,031.290	88,032.920
Entropy	—	0.925	**0.917**	0.935	0.925
BLRT	—	<0.001	**<0.001**	<0.001	0.371
LMRT	—	<0.001	**<0.001**	<0.001	0.374
Group sizes (%)					
C1	100%	31.660%	**42.259%**	3.417%	3.347%
C2	—	68.340%	**26.569%**	26.569%	11.994%
C3	—	—	**31.172%**	45.397%	20.502%
C4	—	—	—	24.616%	42.538%
C5	—	—	—	—	21.618%

*Note:* Bold values indicate the selected three‐profile model.

Abbreviations: AIC, Akaike information criterion; BIC, Bayesian information criterion; aBIC, sample‐size‐adjusted Bayesian information criterion; BLRT, bootstrapped likelihood ratio test; LMRT, Lo–Mendell–Rubin adjusted likelihood ratio test.

### 3.3. Demographic and Academic‐Related Characteristics of Each Profile

Univariate analysis revealed significant differences (*p* < 0.05) among the three latent profiles with respect to gender, age, only child, monthly household income, clinical practice, family atmosphere, nursing interest, personality, CT, and physical activity. Detailed results are presented in Table 1.

### 3.4. Predictor of Latent Profile Membership

Multinomial logistic regression analysis was conducted with the low alexithymia group serving as the reference category to identify predictors of alexithymia profiles (Table 3). For the moderate alexithymia group, several factors were associated with decreased odds of membership relative to the low alexithymia group: being male (OR = 0.402, *p* < 0.001), having a monthly household income ≤3000 RMB (OR = 0.489, *p* = 0.006), and having no clinical practice experience (OR = 0.655, *p* = 0.027). Conversely, students with an introverted personality (OR = 2.275, *p* < 0.001), a neutral personality (OR = 1.505, *p* = 0.024), and those who disliked their nursing major (OR = 2.857, *p* = 0.001) demonstrated significantly increased odds of belonging to the moderate alexithymia group compared to the low alexithymia group. For the hyperalexithymia group, nursing students with a history of CT (OR = 7.679, *p* = 0.006) had substantially higher odds of membership compared to the low alexithymia group.

**Table 3:** Multiple logistic regression analysis of influencing factors of alexithymia among undergraduate nursing students (*N* = 1434).

Variables	Moderate alexithymia vs. low alexithymia					Hyperalexithymia vs. low alexithymia				
	*β*	SE	OR	95% CI	*p*	*β*	SE	OR	95% CI	*p*
Gender (reference: female)										
Male	−0.911	0.200	0.402	0.272–0.595	<0.001	−0.204	0.169	0.815	0.586–1.135	0.226
Age (reference:>23 years)										
≤20 years	−0.446	0.329	0.640	0.336–1.219	0.174	0.032	0.324	1.033	0.548–1.948	0.920
21–23 years	−0.021	0.283	0.979	0.563–1.704	0.941	−0.080	0.289	0.923	0.524–1.625	0.780
Only child (reference: no)										
Yes	−0.106	0.168	0.900	0.647–1.251	0.529	−0.434	0.167	0.648	0.468–0.898	0.009
Monthly household income (reference: ≥9001)										
≤3000	−0.715	0.258	0.489	0.295–0.811	0.006	−0.360	0.234	0.698	0.441–1.104	0.124
3001–6000	−0.257	0.188	0.773	0.535–1.118	0.172	0.125	0.178	1.134	0.799–1.608	0.482
6001–9000	−0.143	0.194	0.867	0.593–1.267	0.461	−0.101	0.191	0.904	0.621–1.315	0.598
Clinical practice (reference: yes)										
No	−0.423	0.191	0.655	0.451–0.952	0.027	−0.142	0.191	0.867	0.596–1.262	0.457
Personality (reference: extroverted)										
Introverted	0.822	0.229	2.275	1.451–3.567	<0.001	0.092	0.212	1.096	0.724–1.661	0.664
Neutral	0.409	0.181	1.505	1.056–2.144	0.024	−0.157	0.150	0.855	0.637–1.147	0.295
Nursing interest (reference: like very much)										
Dislike	1.050	0.323	2.857	1.518–5.379	0.001	−0.456	0.274	0.634	0.370–1.084	0.096
Neutral	0.659	0.291	1.933	1.093–3.419	0.024	−0.067	0.211	0.936	0.618–1.415	0.752
Family atmosphere (reference: authoritarian)										
Highly democratic	0.970	0.352	2.637	1.322–5.261	0.006	0.074	0.254	1.077	0.655–1.770	0.771
Moderately democratic	0.662	0.346	1.939	0.984–3.820	0.056	0.035	0.239	1.035	0.648–1.654	0.885
Childhood trauma (reference: no)										
Yes	—	—	—	—	—	2.039	0.746	7.679	1.780–33.130	0.006
Physical activity (reference: high physical activity)										
Low physical activity	−0.027	0.240	0.973	0.608–1.557	0.910	0.204	0.221	1.226	0.795–1.890	0.356
Moderate physical activity	0.522	0.259	1.686	1.014–2.803	0.044	0.236	0.248	1.267	0.778–2.061	0.341

### 3.5. Network Analysis Across Latent Profiles

Figures 2 and 3 present the network structures and node strength centrality of the four alexithymia dimensions and their related factors, respectively. The network for the low alexithymia profile is shown in Figure 2A. In this profile, the strongest positive connections were identified between externally oriented thinking and difficulty identifying feelings (TAS IV–TAS II, edge weight = 0.36), as well as between difficulty describing feelings and lack of fantasy (TAS I–TAS III, edge weight = 0.31). As shown in Figure 3, difficulty identifying feelings (TAS II) exhibited the highest strength centrality (strength = 0.997). As shown in Figure 4A, the weights of several edges are concentrated around 0, indicating the absence of strong connections between these nodes. However, the distribution on the right side (positive value range) reveals positive associations among certain nodes. As illustrated in Figure 5A, Expected Influence, Closeness, and Strength demonstrate extremely high stability, whereas betweenness exhibits relatively poor stability.

**Figure 2:**
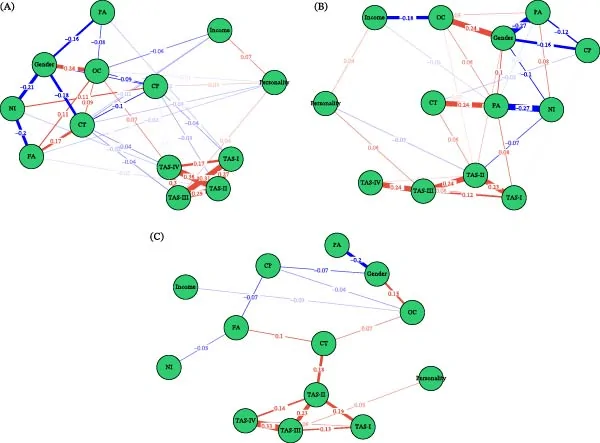
The networks of the three latent profiles. (A) Low alexithymia group. (B) Moderate alexithymia group. (C) Hyperalexithymia group. TAS‐I: difficulty describing feelings; TAS‐II: difficulty identifying feelings; TAS‐III: lack of fantasy; TAS‐IV: externally‐oriented thinking. In the network diagram, red and blue edges represent positive and negative partial correlations, respectively. Furthermore, the thickness of the lines corresponds to the magnitude of the edge weights. CP, clinical practice; CT, childhood trauma; NI, nursing interest; OC, only child; PA, physical activity.

**Figure 3:**
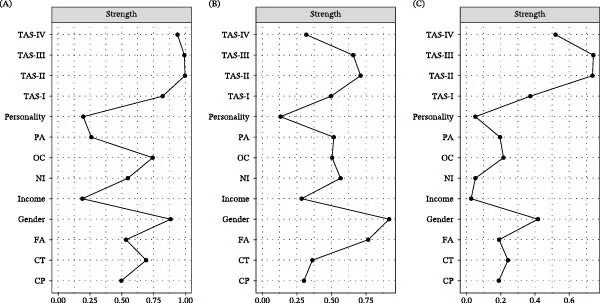
The strength centrality index of the networks of the three latent profiles. (A) Low alexithymia group. (B) Moderate alexithymia group. (C) Hyperalexithymia group.

**Figure 4:**
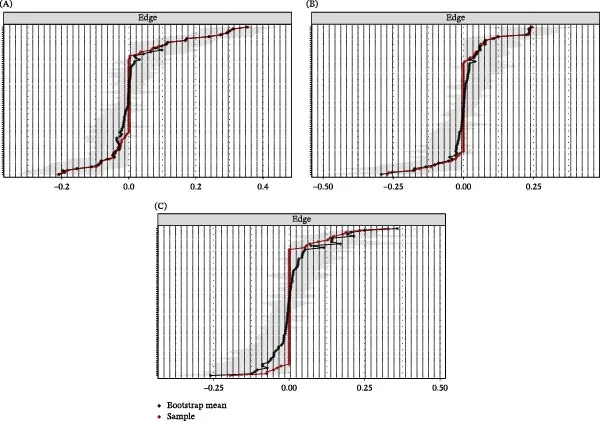
Bootstrapped confidence intervals of edge weights for the networks of the three latent profiles. (A) Low alexithymia group. (B) Moderate alexithymia group. (C) Hyperalexithymia group.

**Figure 5:**
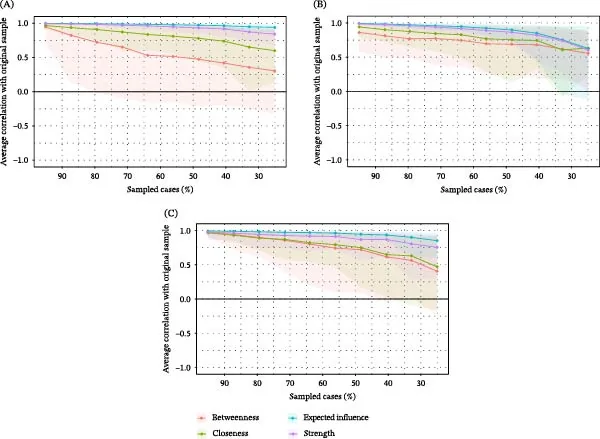
Case‐dropping bootstrap stability analysis of centrality indices for the networks of the three latent profiles. (A) Low alexithymia group. (B) Moderate alexithymia group. (C) Hyperalexithymia group.

The network structure for the moderate alexithymia group is depicted in Figure 2B. Within this network configuration, notable positive edge weights were observed between externally oriented thinking and lack of fantasy (TAS IV‐TAS III, edge weight = 0.24), in addition to a positive association connecting lack of fantasy with difficulty identifying feelings (TAS III‐TAS II, edge weight = 0.24). With respect to external correlates, nursing interest showed an inverse relationship with difficulty identifying feelings (TAS II) (edge weight = −0.07), while family atmosphere demonstrated a positive association with difficulty describing feelings (TAS I, edge weight = 0.08). As shown in Figure 3, strength centrality metrics indicated that difficulty identifying feelings (TAS II) constituted the most influential node in this subgroup’s network, exhibiting the highest strength value (strength = 0.711). As shown in Figure 4B, the weights of several edges are concentrated around 0, with their confidence intervals including 0. However, some edges in the positive (right) and negative (left) regions extend beyond the confidence intervals, indicating significant positive or negative associations between specific nodes. As illustrated in Figure 5B, expected influence and strength maintain high average correlations even after dropping a substantial proportion of cases, demonstrating extremely high stability. In contrast, although the curves for closeness and betweenness drop relatively rapidly, their stability remains greater than 0.5.

Figure 2C presents the network structure of the hyperalexithymia group. The strongest positive association was observed between externally oriented thinking and lack of fantasy (TAS IV–TAS III, edge weight = 0.33), followed by the association between difficulty identifying feelings and lack of fantasy (TAS II–TAS III, edge weight = 0.23). Among the external variables, CT was positively associated with Difficulty Identifying Feelings (TAS II, edge weight = 0.18). As shown in Figure 3, lack of fantasy (TAS III) exhibited the highest strength centrality (strength = 0.741). As shown in Figure 4C, the weights of most edges are concentrated around 0, with wide confidence intervals that include 0. This indicates that the connections between most nodes in the network are weak or nonsignificant. However, in the right (positive) and left (negative) regions, the weights of some edges deviate significantly from 0, with narrow confidence intervals that do not include 0, suggesting significant positive or negative associations between specific nodes. As illustrated in Figure 5C, the expected influence and strength demonstrate extremely high stability. In contrast, the curves for closeness and betweenness drop relatively rapidly and fall below 0.5, indicating poor stability for these two indices.

## 4. Discussion

By applying LPA, the present study identified distinct latent profiles of alexithymia among undergraduate nursing students, highlighting individual differences in alexithymic characteristics. These findings further support the conceptualization of alexithymia as a multidimensional and heterogeneous psychological construct rather than a uniform or unidimensional trait. According to the theoretical model proposed by Taylor et al. [31], alexithymia encompasses several interrelated characteristics, including difficulty identifying feelings, difficulty describing feelings, limited fantasy, and externally oriented thinking. These characteristics may manifest to varying degrees across individuals. Therefore, the latent profiles identified in this study not only distinguish different levels of alexithymia among nursing students but also provide further insight into individual differences in emotional functioning.

Using this person‐centered analytical approach, three distinct latent subgroups were identified: the Low Alexithymia group (42.259%), the moderate alexithymia group (26.569%), and the hyperalexithymia group (31.172%). These findings may be partly explained by Lane and Schwartz’s Levels of Emotional Awareness theory [32], which describes emotional awareness in terms of different levels of cognitive processing, ranging from bodily sensations to complex emotional representations. Accordingly, the identified latent profiles may be associated with differences in individuals’ abilities to identify, understand, and express emotions. Nursing students with higher levels of alexithymia may experience greater difficulty accurately recognizing their emotional states and expressing their emotional experiences verbally. Such difficulties may also affect their emotional regulation and interpersonal interactions. Furthermore, these findings are consistent with previous epidemiological evidence suggesting that nursing students may be a population relatively susceptible to alexithymia [13]. Previous studies have reported associations between higher levels of alexithymia and psychological health problems, including emotional distress, depression, and anxiety symptoms [33]. Notably, nursing education requires students not only to acquire professional knowledge and clinical skills but also to understand patients’ emotions, communicate effectively, and establish positive nurse–patient relationships. Nursing students who experience difficulties identifying and expressing emotions may find it challenging to accurately interpret emotional information and respond appropriately when caring for patients experiencing pain, anxiety, or other complex emotions. Therefore, addressing alexithymic characteristics among nursing students may be important not only for mental health education but also for supporting their clinical communication skills and future professional adaptation. The distinct latent profiles identified in this study also provide preliminary insights into the development of stratified educational strategies. For students in the hyperalexithymia group, nursing educators may consider placing greater emphasis on emotional awareness training, reflective learning, and simulation‐based teaching to help students improve their ability to identify, understand, and express emotions. For those in the moderate alexithymia group, educational activities could focus on emotion identification and labeling, emotional expression, and interpersonal communication skills. Group discussions, role‐playing, and clinical case analyses may provide additional opportunities for emotional interaction and feedback. For students in the low alexithymia group, emotional regulation and communication skills training could be integrated into routine nursing curricula to support the continued development of emotional competence. Previous systematic reviews have suggested that mindfulness‐based interventions and certain psychological interventions may help reduce alexithymic characteristics [34]. In addition, research has indicated that emotional intelligence training may improve emotional intelligence and communication skills [35]. Therefore, nursing education institutions may consider incorporating emotional regulation training, psychological support, and communication skill development into existing curricula while providing differentiated support according to the students’ individual needs.

The elevated TAS‐IV (externally oriented thinking) and TAS‐III (lack of fantasy) scores observed in the moderate alexithymia and hyperalexithymia groups may be related to the specific professional demands of nursing education. Clinical practice require nursing students to interact frequently with patients and healthcare professionals from diverse sociocultural backgrounds, which may encourage greater attention to external information and clinical tasks. As future nursing professionals, students are expected not only to collaborate with physicians but also to understand the emotional experiences of patients and their families. These multiple communication demands may direct students’ attention toward external stimuli and routine clinical activities, potentially reducing opportunities to reflect on and process their own emotional experiences [36]. Furthermore, in clinical settings that emphasize professional conduct, sound clinical judgment, and efficiency, some nursing students may feel the need to maintain a calm and composed professional image while limiting the expression of personal emotions. Over time, limited opportunities for emotional expression and reflection may discourage students from attending to their internal emotional experiences and may be associated with externally oriented thinking and reduced fantasy capacity. Notably, externally oriented thinking and limited fantasy capacity may also have implications for nursing students’ empathy and clinical communication skills. Students who experience difficulties recognizing and expressing emotions may find it challenging to interpret patients’ verbal and nonverbal expressions of distress, such as fear and anxiety, and respond appropriately to their emotional needs. These difficulties may contribute to more task‐oriented nurse–patient interactions, potentially creating challenges in establishing therapeutic relationships based on trust and mutual understanding [37]. Consequently, patients may feel that their emotional needs are insufficiently acknowledged, which could negatively affect their care experiences and satisfaction. Based on these findings, nursing educators may consider implementing more targeted approaches to develop students’ emotional competencies. To address externally oriented thinking, emotional awareness training, reflective journaling, and clinical case discussions could be used to encourage students to attend to their internal emotional experiences and improve their ability to identify and express emotions. For students with limited fantasy capacity, expressive writing, art‐based activities, and simulation‐based learning could be incorporated into nursing education to provide opportunities for exploring and expressing internal emotional experiences [16, 38]. Integrating emotional competence development with clinical communication training may therefore represent a promising educational approach to addressing alexithymic characteristics among nursing students. However, the effectiveness of these strategies requires further evaluation through intervention studies specifically involving nursing students.

Our analysis indicated that female nursing students were more likely to be classified into the moderate alexithymia subgroup. This finding diverges from those of previous studies [39, 40]. This discrepancy may be understood in the context of gender‐specific socialization patterns and emotional processing tendencies. Females are generally more prone to internalizing psychological distress. In contrast to males, whose emotional and behavioral issues may attenuate over time, females often adopt a more restrained style of emotional expression, tending to conceal their emotions and preferences and sometimes even expecting others to intuitively perceive their internal states. Consequently, this emotional processing pattern may be accompanied by the long‐term accumulation of unresolved emotional experiences [41], which could reflect the higher prevalence of alexithymia observed among female nursing students. Furthermore, a lack of professional interest showed a significant association with membership in the moderate alexithymia subgroup. Undergraduate nursing students who hold a positive attitude and genuine passion for the nursing profession often report a stronger sense of professional dignity and honor. Those who choose nursing out of intrinsic interest tend to perceive a heightened sense of responsibility and mission [13]. Compared to their peers with lower interest, these students generally demonstrate greater attentiveness to practical employment market demands and the necessity of acquiring relevant competencies. When confronted with academic or clinical challenges, they often display a greater capacity to channel stress into motivation, a tendency that aligns with higher emotional expressivity and coping resilience. Collectively, these associations highlight the importance of directing targeted support toward female students and those with lower professional interest [42]. Interventions aimed at fostering adaptive career perspectives and strengthening interpersonal communication skills may help reduce the prevalence of alexithymia and contribute to the psychological well‐being of the future nursing workforce.

Our findings underscore a significant positive correlation between CT and alexithymia, corroborating prior research which indicates that more severe childhood maltreatment or neglect is associated with greater deficits in identifying and describing emotions, a pattern that may foster an externally oriented cognitive style [4]. This result provides further evidence that childhood abuse and neglect are linked to long‐term adverse outcomes in personality and psychological functioning in adulthood [43]. Furthermore, this relationship can be interpreted through the lens of the emotional processing model. Individuals with a history of CT often adopt avoidant emotional processing strategies as a defense mechanism against overwhelming negative emotions linked to traumatic memories [44]. While this avoidance may offer short‐term relief from distress, its chronic use tends to be accompanied by unconscious emotional disconnection and suppression. Consequently, this process could hinder the development of skills necessary for recognizing internal emotional states and articulating feelings, potentially reinforcing the cognitive patterns characteristic of alexithymia [43]. Nursing educators and relevant departments of schools should pay close attention to nursing students with CT, promptly understand and monitor their psychological changes, and use professional educational methods to provide timely psychological counseling to address the impact of CT and potentially help alleviate their alexithymia.

Network analysis provides a useful framework for examining the interrelationships among psychological characteristics and identifying highly central nodes. In the present study, TAS‐II (difficulty identifying feelings) and TAS‐III (lack of fantasy) exhibited the highest strength centrality in both the moderate alexithymia and hyperalexithymia subgroups, suggesting that these two dimensions were strongly connected to other alexithymic characteristics. Difficulty identifying feelings reflects limitations in recognizing and distinguishing internal emotional states, whereas a lack of fantasy may reflect reduced engagement in imaginative activities and internally generated emotional experiences. The prominence of these dimensions within the networks suggests that difficulties in emotional awareness and internal emotional representation may be particularly relevant to the interconnected structure of alexithymic characteristics among nursing students. From a network perspective, highly central nodes have been proposed as potential intervention targets as changes in these nodes may be associated with changes in other connected characteristics [45, 46]. These findings may also be understood in the context of the academic and clinical demands of the nursing education. Undergraduate nursing students often face substantial academic workloads, clinical responsibilities, and interpersonal demands, which may contribute to psychological stress and emotional exhaustion [37, 47]. Under sustained pressure, students may devote greater attention to completing academic and clinical tasks, leaving fewer opportunities to reflect on and process their emotional experiences. This may be particularly relevant to difficulty identifying feelings as emotional awareness requires individuals to attend to and interpret their internal emotional states. Similarly, limited opportunities for emotional reflection and imaginative engagement may be associated with a reduced fantasy capacity. In light of these findings, our study provides preliminary implications for potential targeted measures, such as incorporating emotion management and psychological counseling into nursing education curricula. By fostering a supportive psychological environment and encouraging students to express their emotions in healthy and adaptive ways, educators may help promote students’ psychological well‐being and potentially alleviate the severity of alexithymia. Notably, alexithymia may also represent a potential vulnerability pathway linking psychological distress and difficulties in emotion regulation to more serious mental health outcomes, including suicidal ideation among university students [48]. Therefore, greater attention should be given to the early identification of alexithymia, psychological distress, and related internalizing symptoms in university settings, alongside targeted interventions aimed at enhancing emotional awareness and expression. Nevertheless, the effectiveness of these interventions and optimal intervention strategies remain to be established through future experimental or longitudinal studies.

## 5. Limitations

Despite the valuable insights yielded by this study, several limitations warrant consideration. First, although recruiting students from 10 universities enhanced the sample diversity, the reliance on a convenience sampling method may have introduced selection bias and compromised the representativeness of the participants. Moreover, the findings are situated within a specific cultural and educational context, which may limit their generalizability to nursing students in other cultural settings. Future investigations should employ stratified or cluster sampling across diverse regions to enhance external validity. Second, the cross‐sectional design precludes the establishment of causal relationships between alexithymia and its associated factors. To address this, longitudinal studies are essential to map temporal networks and explore the dynamic evolution of alexithymic traits over time. Finally, the exclusive use of self‐report measures makes the data susceptible to reporting biases, such as social desirability and recall biases, which may compromise data accuracy. Subsequent research should incorporate multi‐method assessments, including objective physiological markers or informant reports, to triangulate the findings and mitigate these biases.

## 6. Conclusions

Collectively, the present findings delineate three unique latent profiles of alexithymia within the undergraduate nursing student population, highlighting the necessity for precision‐oriented psychological support. Specifically, female gender, a history of CT, and low professional interest were identified as the primary risk factors. Moreover, the network topology illustrated heterogeneous central nodes across these distinct subgroups. Ultimately, these insights advocate for the implementation of customized intervention frameworks by nursing educators, which must be meticulously aligned with the specific vulnerabilities and core deficits characterizing each profile.

## Author Contributions

**Ying Li**: conceptualization, methodology, data curation, writing – original draft, writing – review and editing. **Songwei Zhong:** data curation. **Meng Xiang:** methodology.

## Funding

The authors declare that no financial support was received for the research, authorship, and/or publication of this article.

## Disclosure

The authors have nothing to report.

## Ethics Statement

This study was approved by the Ethics Committee of Jishou University (Approval Number JSDX‐2025‐0131). Written informed consent was obtained from all participants before participation in the study.

## Conflicts of Interest

The authors declare no conflicts of interest.

## Data Availability

The data that support the findings of this study are available from the corresponding author upon reasonable request.
